# Metagenomics of pigmented and cholesterol gallstones: the putative role of bacteria

**DOI:** 10.1038/s41598-018-29571-8

**Published:** 2018-07-25

**Authors:** S. H. Kose, K. Grice, W. D. Orsi, M. Ballal, M. J. L. Coolen

**Affiliations:** 1 School of Molecular and Life Sciences, Curtin University, Perth, WA 6102 Australia; 2 WA-Organic and Isotope Geochemistry Centre, School of Earth and Planetary Science, Curtin University, Perth, WA 6102 Australia; 3 Department of Earth and Environmental Science, Paleontology and Geobiology, Ludwig-Maximilians-Universität München, 80333 Munich, Germany; 4 GeoBio Centre LMU, Ludwig-Maximilians-Universität München, 80333 Munich, Germany; 5 Fiona Stanley Hospital, 11 Robin Warren Dr, Murdoch, 6150 WA Australia; 6 St John of God Murdoch Hospital, Barry Marshall Parade, Murdoch, 6150 WA Australia

**Keywords:** Bacterial genes, Gall bladder

## Abstract

There is growing evidence for bacteria playing a role in the pathogenesis and formation of pigmented gallstones from humans. These studies mainly involved cultivation of gallstone-associated bacteria and 16S rRNA profiling, providing an indirect link between processes involved in gallstone formation by the bacteria *in-situ*. Here, we provide functional metagenomic evidence of a range of genes involved in bile stress response, biofilm formation, and anaerobic energy metabolism by Gram-negative *Klebsiella* in pigmented gallstones from a 76-year-old male patient. *Klebsiella* was also present in one cholesterol-type stone in a 30-year-old female patient who had additional cholesterol gallstones characterised by Gram-positive bacteria. Pigmented stones further revealed a predominance of genes involved in carbohydrate metabolism, whilst cholesterol stones indicated a profile dominanted by protein metabolism possibly reflecting known chemical differences between Gram-negative and Gram-positive biofilm matrices. Archaeal genes were not detected. Complementary carbon and hydrogen isotopic analyses of cholesterol within the patients’ stones revealed homogeneity, suggesting a common diet or cholesterol biosynthesis pathway that has little influence on microbial composition. This pilot study provides a framework to study microbial processes that play a potential role in gallstone formation across markedly different types of stones and patient backgrounds.

## Introduction

The focus on bacteria and its role in gallstone pathogenesis began most notably in 1966 by Maki^1^, and furthered by Stewart *et al*.^2,3^. In those studies, bacteria were suggested to play a causal role in the pathogenesis of pigmented and the pigmented portion of mixed stones only, as cholesterol stones rarely exhibited bacterial signatures^2^. A conclusive definition of gallstone types and their bacterial compositions are yet to be determined due to the complex nature of gallstones. However, researchers do generally group the stone types into cholesterol (predominantly composed of cholesterol, that may have pigmented centres), pigmented (predominantly composed of the bile pigment bilirubin, derived from the break down of aged red blood cells by the liver giving its brown colour), mixed (a compositional mixture of the cholesterol and pigmented) and black (assumed to be pigmented but with a black ‘volcanic glass’ type appearance). For a detailed discussion on stone types and compositions see Stewart *et al*.^4^. A further study restricted gallstone pathogenesis *via* bacterial action to only brown pigmented stones, ruling out cholesterol, mixed pigmented and black pigmented gallstones altogether^5^. Subsequently, bacterial studies were focused on pigmented stone formation with the dominant theory behind the mechanism of formation for this type of stone through the activity of bacterially produced ß-glucuronidase^1^. ß-glucuronidase was observed to be the deconjugating factor that led to the precipitation of calcium bilirubinate crystals, with these crystals conjugated by an anionic glycoprotein (i.e. sodium alginate) leading to the agglomeration of the bilirubinate calcium crystals into macroscopic stones^1^. However, Maki’s findings related to *Escherichia coli* (a known producer of ß-glucuronidase), and did not explore non-ß-glucuronidase producing bacterial species that are often found in gallstone studies^3^. Biofilm formation as an alternative mechanism for the role of bacteria in pigmented stone formation was initially proposed by Stewart *et al*.^2^. In that culture-based study, the biofilm product glycocalyx (an anionic glycoprotein), was suggested to be the central agglomerating factor, with ß-glucuronidase having a comparatively minor role. *Klebsiella*, *Enterococcus*, *Enterobacter*, *E*. *coli* and *Psuedomonas aeruginosa*, were reported to be the most prevalent cultured bacteria across 61 stones (predominantly pigmented and mixed stones), with *P*. *aeruginosa* the greatest biofilm producer of the group after *Citrobacter freundii*^3^. A more recent study involving mice as a host identified *Salmonella*, *via* scanning electron microscopy (SEM) and culture analysis, as a primary producer of biofilms on the surface of cholesterol gallstones^6^. However, only up to 1% of bacteria in complex environmental samples can generally be brought into culture and provides a biased view into the relative abundance of the total species present. A recent cultivation-independent investigation of the microbial composition of 27 cholesterol gallstones using high-throughput 16S rRNA profiling identified a predominance of *Enterobacteriaceae* and *Ruminococcoceae* and to a lesser extent, *Bacteroidales*, *Lactococcus*, *Enterococcus and Clostridiales* within the analysed gallstones^7^. However, 16S rRNA profiling does not provide information on the role the identified bacteria play in the formation of gallstones and what mechanisms they possess to survive in the human gallbladder.

In this study we analysed functional metagenomes^8^ to investigate the diversity and metabolic potential of microbial communities in pigmented *vs*. cholesterol stones, and whether they possess genes involved in the formation of biofilms or other processes including bile resistance that could lead to gallstone formation. The sequencing of functional metagenomes was furthermore used to provide parallel information on the functional and/or taxonomic diversity of all domains of life not limited to bacteria (e.g. fungi, archaea, and viruses) that may be present and underexplored in gallstones^9,10^. To the best of our knowledge, the only other related shotgun metagenomics study was perfomed on human bile samples^11^. However, it has been shown that bacterial biofilms may persist on the surface of gallstones, even when the patient’s bile is culture negative^12^.

We complement the shotgun metagenomics analysis with compound specific isotopic analysis (CSIA) of cholesterol in the gallstones - namely carbon (δ ^13^C) and hydrogen (δD) isotopes to ascertain possible dietary or exogenous environmental factors that may be associated with or divergent from the bacteria identified in this study.

## Results and Discussion

Gallstones were collected from the gallbladders of a 76-year old male patient (PM1) with pigmented type gallstones (n = 4) and one 30-year old female patient (CF4) with cholesterol type gallstones (n = 4) (Table 1). Both patients were diagnosed with gallstone disease (cholelithiasis) by ultrasound imaging and computed tomography scanning at the Fiona Stanley Hospital and St John of God Hospital, Murdoch, WA, Australia. Neither patient had been given a course of antibiotics for treatment. Pigmented gallstones contained 57.2 ± 36.6 ng per stone genomic DNA while the DNA content in the cholesterol gallstones was 16.5 ± 10.9 ng per stone. This DNA served as template for the construction of the metagenomics libraries discussed below.

**Table 1:** Patient Information.

SampleID	Age	Sex	BMI	Antibiotics	Type	Size	No.	Site
PM1	76	M	24	Nil	Pigmented	Small 0.5–1 cm	4	FSH
CF4	30	F	40.5	Nil	Cholesterol	Small 0.5–1 cm	4	SJOG

### Metagenomics

Metagenomic analysis revealed distinct differences between the microbial diversity of the two patients. The following section focuses on the taxonomic diversity and gene functions potentially associated with gallstone pathenogensis and formation.

**Figure 1:**
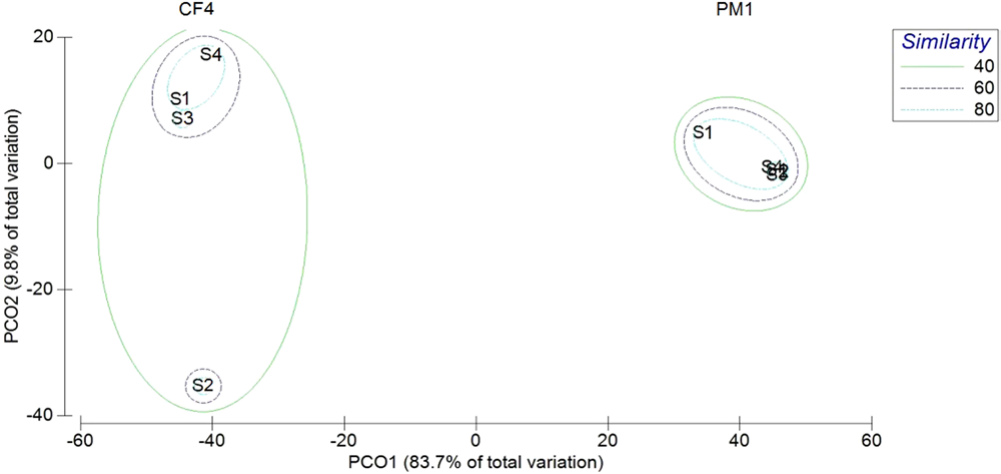
Principle coordinate analysis (PCoA) ordination of Bray-Curtis similarity (square root transformed) between total bacterial genera in the four gallstones of patients PM1 and CF4. Shown is the first two principle coordinate axes, which combined explain 93.5% of the variation between the patients. Coloured ellipses signify the percentage of similarity between the patients’ native stones.

Principle component analysis (PCoA; Fig. 1) on open reading frames (ORFs) revealed that the first component explains 83.7% of the variance in microbial diversity in the analysed gallstones. The second component explains 9.8% of the variation, with both components combined accounting for 93.5% of the variation between the two patients. Only 5.5% of the variation remains unexplained. The microbial composition between the four stones found in patient PM1 showed a high level of similarity at 80%. A 60% similarity in microbial diversity was observed between stones 1, 3 and 4 of patient CF4. Stone 2, however, only shared 40% similarity to the group (Fig. 1).

### Taxonomic affiliation

**Figure 2:**
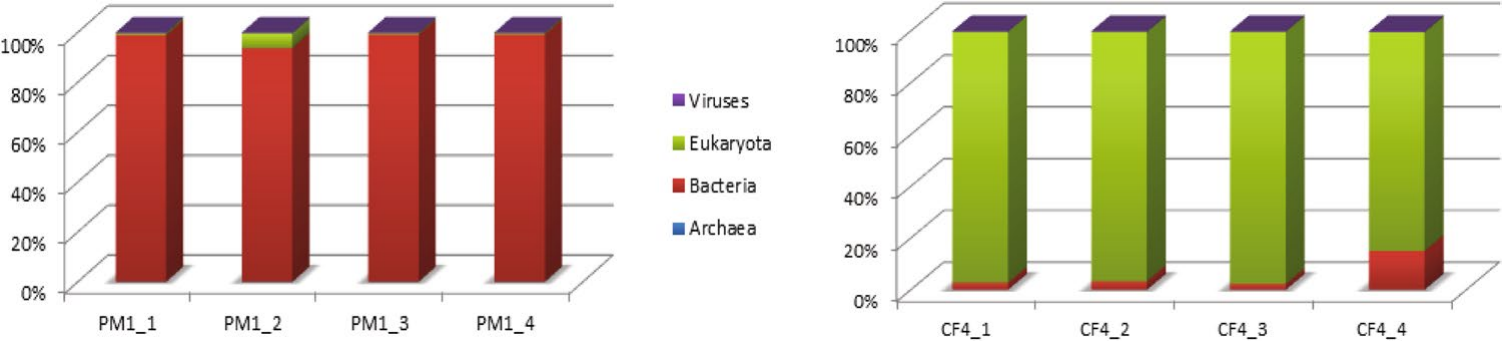
Relative abundance of bacteria, archaea, eukaryotes and viruses recovered from metagenomes in patients PM1 (left) and CF4 (right).

Taxonomic analysis of the ORFs of 4 replicate pigmented gallstones of patient PM1 revealed a predominance of bacteria (97.65%) while no archaeal ORFs were recovered (Fig. 2). Eukaryotes comprised 1.98% of the ORFs with the majority (80.49%) of human origin. A small proportion of the ORFs were of viral origin (0.3%) and misidentified reads (0.07%).

**Figure 3:**
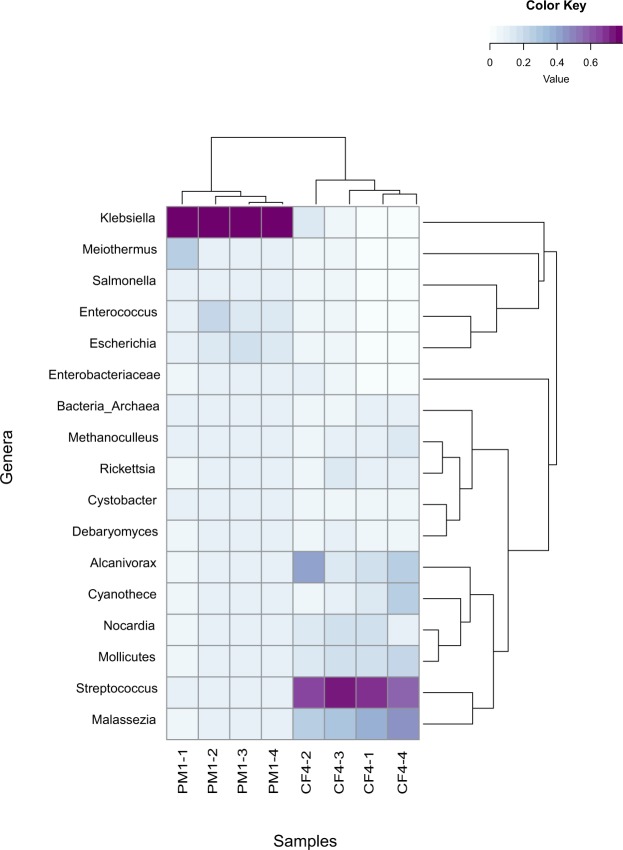
Heatmap with the major bacterial genera identified in the gallstones (n = 4) of patients PM1 (pigmented) and CF4 (cholesterol). The color key shows the relative abundance of the genera in the gallstones. The dendrograms illustrate the relationship between samples showing that the distribution of genera is relatively similar between replicate stones, but greatly differ between the two patients.

Taxonomic analysis of bacterial ORFs comprising >1% of the total community revealed that Gram-negative *Klebsiella* was the most abundant genus in PM1 (69–79%), followed by *Enterococcus* (3.7–13.6%), *Escherichia* (2.9–8.0%), *Salmonella* (1.7–2.2%), and *Enterobacter* (1.1–1.3%). *Meiothermus* was abundant in stone 1 (17.4%), but was not detected in the other stones of PM1 (Fig. 3).

Metagenomic profiling of 4 replicate cholesterol gallstones of patient CF4 revealed a predominance of eukaryotes (96.05%) with the majority (98.5%) stemming from the patient or comprising human cells, with others (1.46%). Bacteria comprised only 3.6% of the ORFs. In agreement with the metagenomes of PM1 stones, a small portion of the ORFs were of viral origin (0.3%) and misidentified reads (0.05%), while no archaeal ORFs were recovered (Fig. 2).

Taxonomic analysis of bacterial ORFs comprising >1% of the total bacterial community revealed that Gram-positive *Streptococcus* was the most abundant genus within all 4 stones in CF4 (38.4–54.7%) (Fig. 3), followed by *Alconivorax* (6.4–27.8%), *Mollicutes* (6.3–15.1%), *Nocardia* (7.0–13.0%), *Methanoculleus* (5.5–8.1%), *Ricketssia* (4.9–6.9%), and *Cystobacter* (2.7–4.1%). Stone 2 only showed 40% similarity with the other three stones as it did not contain *Nocardia*, *Methanoculleus*, *Ricketssia*, and *Cystobacter*. It was, however, the only stone to exhibit *Klebsiella* (6.8%) and other *Enterobacteriaceae* (4.5%). *Geobacillus* was found only in stones 3 and 4 (1.1–1.2%).

As mentioned earlier, a consensus is yet to be drawn on what type of bacterial communities are common to pigmented and cholesterol gallstones^7^. This is in part due to the complex and unique interplay between a patient’s health history, microbiome, environment, and a predominance of culture studies in which total community resolution is limited^7^. Nevertheless, in our study, patient PM1’s pigmented stone bacterial community do approximately align with a previous culture analysis in which *Klebsiella*, *Enterococcus*, *Enterobacter*, *E*. *coli* and *Psuedomonas aeruginosa* were found to be the most prevalent genera across 61 pigmented and mixed gallstones^3^.

Less is known about microbial compositions in cholesterol gallstones, due to their propensity to have low amounts of bacterial DNA. However, this assertion may be due to the limitation of culture studies, as a recent investigation of the microbial composition of 27 cholesterol gallstones using high-throughput 16S rRNA profiling identified *Enterobacteriaceae* to be abundant^7^, whereas the less abundant genera differed from those present in patient CF4 in our study.

Quantitative PCR revealed a two orders of magnitude higher bacterial 16S rRNA copy number (~ 9.9 × 10^6^ ± 4.6 × 10^6^) in pigmented gallstones of PM1 compared to the cholesterol-type gallstones of CF4 (3.1 × 10^4^ ± 2.0 × 10^4^ copies per stone). The results of this cultivation-independent molecular quantification approach are consistent with previous culture studies that report a greater microbial biomass in pigmented *vs*. cholesterol stones^1–3,13^. We provide a conservative estimate of bacterial cell numbers (1.2 × 10^6^ ± 5.8 × 10^5)^ bacterial cells per stone in PM1 and 7.7 × 10^3^ ± 5.0 × 10^3^ cells per stone in CF4) based on known number of ribosomal operons in the most abundant genera found in PM1 (*Klebsiella* with 8 rRNA operons^14^) and CF4 (*Streptococcus* with 4 rRNA operons^15^).

### Functional metagenomic profiling

#### Genes involved in Bile Stress response

Microorganisms have been known to survive and thrive in a range of extreme environments, including the human body where variations in pH, nutrient limitations, low oxygen levels, an established diverse bacterial microbiome, and host immunity responses provide longterm obstacles for survival^16–18^.

The human gallbladder environment poses its own unique challenges. The liver secretes approximately 800–1000 mL of bile into the gallbladder per day^19^. Bile acts as a detergent or emulsification agent for the digestion and absorption of lipids and contains sodium and potassium salts, bile acids (namely chenodeoxycholic acid, cholic acid), cholesterol, phospholipids, and bile pigments such as bilirubin^19^. This environment is made further hostile to bacteria as bile is concentrated 5–10 fold in the gallbladder, with commonly used antibiotic drugs, and heavy metals being secreted into bile as per the liver’s detoxification and enterohepatic cycling processes^16,19^.

The strategies bacteria employ to resist toxic agents such as bile and antibiotics are often complex and involve a variety of methods that include and are not limited to efflux pumps (which pump the toxic agent out of the cell), reduction of cell permeability, enzymatic modification or destruction of toxic agents either within or without the cell wall barrier, and the modification of the toxic agent’s target either *via* genetic mutation, enzymatically, or by presenting an alternate target^20,21^. Bile, as a detergent, and consisting of a variety of toxic agents has been shown to cause membrane perturbations, DNA damage and oxidative stress in bacteria^21^. This is shown to be consistent with our current study where multidrug export efflux pumps, DNA and cell wall repair proteins were identified as important in bile resistance. Of the seventeen taxa in both patients combined that comprised at least 1% of the total sequenced gene pool, we identified six genera that harbour genes involved in bile stress survival, which are linked to the production of biofilms that may be associated with pigmented as well as cholesterol-type gallstone formation (Tables 2, 3 and 4) as discussed in detail below.

**Table 2:** Loci disrupted in bile-sensitive mutants and the functions of gene products.

Genes disrupted in bile-sensitive mutants	Function of gene products/putative function	Reference(s)
**Gram-negative bacteria**		
*Klebsiella*		
*marA*	Multiple antibiotic resistance protein – Regulatory genes	^11,19,20^
*marB*	Multiple antibiotic resistance protein – Regulatory genes	^11,19,20^
*marC*	Multiple antibiotic resistance protein – Regulatory genes	^11,19,20^
*marR*	Regulatory genes	^11,19,20^
*TolC precursor*	Efflux pump function	^17,21^
*dam*	DNA adenine methylase	^24^
*emrE*	Efflux pump –drug resistance	^31^
*emrB*	Efflux pump – drug resistance	^31^
*mdtABCD*	Efflux pump –multi drug resistance	^22,31^
*cmeA*	Efflux pump	^23,31^
*cmeB*	Efflux pump	^23,31^
*sbcC*	Exonuclease	^25^
*MutS*	DNA mismatch repair	^25^
*nifJ homolog*	Pyruvate flavodoxin oxidoreductase	^25^
*yvaG*	3-oxoacyl-acyl-carrier protein reductase	^25^
*dgt homolog*	Deoxyguanosinetriphosphate triphosphohydrolase	^25^
*Escherichia*		
*yvaG*	3-oxoacyl-acyl-carrier protein reductase	^25^
*Shigella*		
*PhoQ*	Regulatory genes	^34^
**Gram-positive bacteria**		
*Enterococcus*		
*emrB*	Efflux pump – drug resistance	^31^
*sbcC homolog*	Exonuclease	^25^
*MutS*	DNA mismatch repair	^25^
*nifJ homolog*	Pyruvate-flavodoxin oxidoreductase	^25^
*yvaG*	3-oxoacyl-acyl-carrier protein reductase	^25^
*dgt homolog*	Deoxyguanosinetriphosphate triphosphohydrolase	^25^

**Table 3:** Promoters, proteins or open reading frames (ORFs) induced by bile and their functions.

ORFs/promotors/proteins induced by bile	Function of gene products/putative function	Reference(s)
**Gram-negative bacteria**		
*Klebsiella*		
*DnaK*	Molecular chaperone	^18,25^
*GroEL/GroES*	Heat shock protein- Molecular chaperone	^18,25^
*Ohr*	Organic hydroperoxide resistance	^11,18,25^
*Hsp20*	Heat shock protein, molecular chaperone	^18^
*ClpB*	ATP-binding chain of an ATP-dependent protease	^18^
*RecR homolog*	Recombinase (DNA repair)	^11,33^
*sodA*	Manganese superoxide dismutase	^18,27^
*BCCP*	Biotin-containing carboxyl carrier protein of acetyl-CoA carboxylase	^18,57^
*CysK homolog*	Putative cysteine synthase	^18,33^
*ORF002*	NADPH dependent aldo or keto-oxidoreductase	^28,29^
*MutB*	Putative Methylmalonyl-CoA mutase	^11,33^
*AspA*	Putative Aspartate ammonia-lyase	^18^
*G6PD*	Glucose-6-phosphate 1-dehydrogenase	^30,32^
*ATPG*	ATP synthase gamma chain	^18,57^
*HemH homolog*	Putative Ferrochelatase, protoheme ferro-lyase	^18,33^
*dmsABC*	Anaerobic dimethyl sulfoxide reductase chain A,B & C	^39–41^
*Escherichia*		
*ORF002*	NADPH dependent aldo or keto-oxidoreductase	^30,32^
*Shigella*		
*ORF001*	NADPH dependent aldo or keto-oxidoreductase	^30,32^
*ORF002*	NADPH dependent aldo or keto-oxidoreductase	^30,32^
*Serratia*		
*ClpB*	ATP Binding Chain on ATP-dependent protease	^18^
**Gram-positive bacteria**		
*Enterococcus*		
*DnaK*	Molecular chaperone	^18,25^
*GroEL/GroES*	Heat shock protein- Molecular chaperone	^18,25^
*Ohr*	Organic hydroperoxide resistance	^11,26^
*Gsp*	General stress protein	^11,18,25^
*clpB*	ATP Binding Chain on ATP-dependent protease	^18^
*sodA*	Manganese superoxide dismutase	^18,27^
*BCCP*	Biotin-containing carboxyl carrier protein of acetyl-CoA carboxylase	^18,57^
*CysK homolog*	Putative cysteine synthase	^18,33^
*OppD homolog*	Oligopeptide transport ATP-binding protein	^18,35^
*G6PD*	Glucose-6-phosphate 1-dehydrogenase	^30,32^
*ATPG*	ATP synthase gamma chain	^18,57^
*Bacillus*		
*OppD homolog*	Oligopeptide transport ATP-binding protein	^18,35^

**Table 4:** Genes associated with biofilm production and their functions/putative functions.

EPS related genes	Function of gene products/putative function	Reference(s)
**Gram-negative bacteria**		
*Klebsiella*		
CsgD	Transcriptional regulator	^39^
*Gsp*	General stress protein 18	^38^
*fim Type I*	Type 1 fimbriae fimA,B,D,E,L,F,G	^12,39^
*fim Type IV*	Type IV fimbrial assembly, ATPase PilB	^12,39^
wza	Polysaccharide export lipoprotein	^12,39^
wzc	Tyrosine-protein kinase	^12,39^
*Ribose ABC Transport System*	Ribose ABC transport system, ATP-binding protein RbsA	^39^
*Ribose ABC Transport System*	Ribose ABC transport system, permease protein RbsC	^39^
*Autoinducer 2*(*AI-2*)	ABC transport system, fused AI2 transporter subunits and ATP-binding component	^39^
*CP4-57- integrase*	putative CP4-57-type integrase	^39^
*Polyphosphate kinase*	Polyphosphate kinase- Biofilm development	^48^
*sugE*	Quaternary ammonium compound-resistance protein	^12^
*ClpX*	ATP-dependent Clp protease ATP-binding subunit	^12^
*RapA*	RNA polymerase associated protein reg. yhcQ, YeeZ	^17^
*LuxR*	Transcriptional regulator	^12^
*CspD*	Cold shock protein CspD	^12^
*Escherichia*		
wzb	Low molecular weight protein-tyrosine-phosphatase	^39^
wzc	Tyrosine-protein kinase	^39^
***Gram-positive bacteria***		
*Enterococcus*		
*RbsA*	Ribose ABC transport system, ATP-binding protein	^39^
*galE*	UDP-glucose 4-epimerase	^41^
*sugE*	Quaternary ammonium compound-resistance protein SugE	^12^
*ClpX*	ATP-dependent Clp protease ATP-binding subunit	^12^
*LuxR*	Transcriptional regulator	^12^
*CspD*	Cold shock protein CspD	^12^

### Gram-negative Bacteria

#### Klebsiella (bile-sensitive genes)

The *marABC* and *marR* operons (for all genes identified see Table 2) are regulatory genes that control multiple antibiotic drug resistance^22^ and have been shown to be activated in the presence of the bile salt deoxycholate, with the level of gene expression dependent on the salts’ concentration^16,23^. The *Tol* protein and derivatives are important in many Gram-negative bacterial transport systems and act as an outer membrane pore function or efflux pump^24^. Mutations in *Tol* genes destabilise the membrane allowing for greater bile salt entry, thereby affecting bile resistance^24^. The *TolC* efflux pump (Table 2), in particular, has been shown to be upregulated in biofilms and is associated with the removal of toxic compounds and antibiotics^21^. The *emrEB*, *mdtABCD*, *cmeAB* genes similarly correspond to efflux pump action and are essential for bile resistance^24^. The *emrEB* multidrug efflux pump systems have been shown to actively efflux the bile salt chenodeoxycholic acid^25^. Furthermore, over-expression of *mdtABCD*, a multidrug resistance efflux pump cluster, leads to increased deoxycholate resistance^26^. The *cmeAB* has been shown to function as a multidrug efflux pump in *C*. *Jejuni* by effectively mediating resistance to bile salts^27^. The *Dam* (DNA adenine methylase) enzyme has been associated with repairing damage to *Salmonella* DNA after bile acid exposure^28^. Similarly, the *sbcC* and *MutS* (DNA repair), *yvaG* (rebuilding the cell membrane after stress), *nifJ* (oxidative response) and *dgt* (dGTP hydrolysis) homologs were associated with DNA and cell wall repair in response to bile stress in *Enterococcus faecelis*^29^.

#### Klebsiella (ORF’s, promotors, proteins induced by bile)

Experiments with bile salt treatments to *Enterococcus faecelis*^16,29^ identified an increased production of a number of stress proteins (*Gsp*). Three of these stress proteins were identified in this study as the *DnaK* and *GroEL/GroES* molecular chaperones^29^ and the organic hydroperoxide resistance protein *Ohr*^16,30^ within *Klebsiella* (Table 3). Further experiments with *Propinobacterium freudenraichii* revealed genes involved in a variety of stress responses (heat, acid, bile salts) termed *GSPs* (General stress proteins), with bile salts in particular associated with oxidative stress responses^31^. A number of these were annotated to *Klebsiella* in this study and include the molecular chaperones *Hsp*2*0* (heat stress), *DnaK*, *GoEL*, *AspA and ClpB* that are associated with acid stresses^31^. *SodA*, an oxidative damage remediation gene, was also identified in this study and has been shown to be involved in stress responses within *Lactobacillus lactis* (oxygen stress), *Bacillus subtilis* (heat, salt and ethanol stresses), and *B*. *cereus* (heat, salt and ethanol stress)^31,32^. Further oxidative damage reduction and remediation proteins identified for *Klebsiella* were *ORF002*, *G6PD*, and the *CysK*, *HemH* homologs. The NADPH dependent aldo or keto-oxidoreductase *ORF002* protein is an important part of the glutathione cellular defense system that is involved in the reduction of oxidative stress caused by reactive oxygen species (ROS) associated with bile^32,33^. The *G6PD* (Glucose-6-phosphate 1-dehydrogenase) protein has been shown to be activated in the presence of ROS, that arise due to stresses such as high levels of salt, and considered vital for cellular redox balance^34,35^. Similarly, the *CysK* (cysteine synthase) and *HemH* (ferrochetalse) homologs have also been shown to be overexpressed when exposed to bile-salt stresses^31,36^. Other acid stress proteins identified were *BCCP* (a biotin containing carboxyl carrier protein) and *ATPG* (ATP synthase gamma chain)^31,37^. The DNA damage repair proteins *MutB* (Methylmalonyl-CoA mutase) and *RecR* (Recombinase) were also identified^16,36^.

Genes encoding the dissimilatory dimethylsulfide reductase A,B and C (dmsABC, Table 3) were retrieved in *Klebsiella*, indicating its capacity for anaerobic metabolism and use of dimethylsulfoxide (DMSO) as a terminal electron acceptor. DMSO respiration is energetically favourable under anaerobic conditions in bacteria that contain this metabolic potential^38^. Further, the dmsABC operons are controlled by the oxidative regulator *fnr*, important for oxidative stress response and anaerobic metabolism in pathogenic bacteria^39–41^. This ability by *Klebsiella* may explain its successful survival and growth in the anoxic conditions present in the human gallbladder and its dominance in the present study. Other gram-negative and gram-postive bacteria identified in this study may be out competed by *Klebsiella* or utilise fermentation for energy metabolism instead, a less efficient form of energy conservation than DMSO^42^.

#### Escherichia (bile-sensitive genes)

Similar to *Klebsiella*, *Escherichia* exhibited the oxido reductase gene *yvaG* or membrane composition and repair protein in our study (Table 2).

#### Escherichia (ORF’s, promotors, proteins induced by bile)

Like *Klebsiella*, *Escherichia* exhibited the NADPH dependent aldo or keto-oxidoreductase *ORF002* protein involved in the reduction of oxidative stress caused by ROS associated with bile (Table 3).

#### Shigella (bile-sensitive genes)

*Shigella* exhibited the *PhoQ* regulatory protein (Table 2). *PhoQ* is closely associated with the *PhoP* regulon. The combined *PhoP-PhoQ* proteins have been associated with various bacteria and their ability to sense and resist bile stress^43^. Bacterial mutants missing *PhoP-PhoQ* were killed at significantly lower concentrations of bile than those with these proteins, and those with *PhoP* alone surving a >60% concentration of bile in lab conditions^43^^.^

#### Shigella (ORF’s, promotors, proteins induced by bile)

Similar to *Klebsiella* and *Escherichia*, *Shigella* exhibited the NADPH dependent aldo or keto-oxidoreductase *ORF001* and *ORF002* proteins involved in bile oxidative stress reduction (Table 3).

#### Serratia (ORF’s, promotors, proteins induced by bile)

Bile sensitive genes for *Serratia* were not identified. However, *Serratia* exhibited the *ClpB* molecular chaperone involved in acid stress reponses (see *Klebsiella* above).

### Gram-postive bacteria

#### Enterococcus (bile sensitive genes)

Similarly to *Klebsiella*, the important proteins for bile resistance in *Enterococcus* were the multidrug export efflux pump system *emrB*, and those associated with DNA and cell wall repair; *sbsC homolog*, *MutS*, *yvaG*, *nifJ* and *dgt* homologs.

#### Enterococcus (ORF’s, promotors, proteins induced by bile)

As per *Klebsiella*, we see the following same bile stress, oxidative stress and DNA repair genes associated with *Enteroccoccus*: *Dnak*, *GroEL/GroES*, *Ohr*, *Gsp*, *clpB*, *sodA*, *BCCP*, *G6PD*, *ATPG* and the *CysK homolog*. The *OppD homolog* (ABC transporter – ATP binding protein), involved in the efflux of bile and antibiotic resistance during biofilm formation, was also present^31,44^.

#### Bacillus (ORF’s, promotors, proteins induced by bile)

Bile sensitive genes for *Bacillus* were not identified in the metagenomes. However, *Bacillus* exhibited the *OppD homolog* as described above for *Enterococcus*.

#### Genes involved in biofilm production

In addition to mediating toxic substances *via* the resistance strategies described above, microorganisms can group together, attach to either living or  non-living surfaces, and form what is called a biofilm^45^. Biofilms comprise a variety of microorganisms enclosed in an extracellular polymeric substance (EPS) or matrix made up of mostly polysaccharides and other environmental specific materials^45^. Biofilm formation has considerable advantages and has shown to protect bacterial communities from UV light, heavy metals, acidity, hydration or salinity changes and host immune responses, including large doses of antimicrobial drugs that would be lethal to the same community if in a planktonic state^21,46^. There are three main stages involved in biofilm formation: initial adherence to a surface, development of a community structure and ecosystem, and eventual detachment^45^. Each stage is a complex process regulated by a variety of genes that are often environmental, bacterial strain or stressor specific^21^. Common mechanisms include the development of curli fimbriae (adherence or attachment mechanisms), and quorum-sensing or cross community communication to coordinate biofilm attachment, development, detachment, and resistance^13,46^. The main resistance mechanisms afforded by the EPS matrix include drug indifference, in which the EPS works as a barrier between the drug and the targeted microbial cell membrane, the allowance of antibiotics to slowly diffused through the EPS, so that time is given for resistance mutations to develop, efflux pumps, and the secretion of periplasmic glutans that keep toxic substances away from intracellular targets^21^. Certain organisms may provide the base biofilm, whilst others live either competitively or symbiotically within it, with environmental and community composition in a state of constant change^46^. As previously reported, biofilms with *Salmonella* were identified through SEM and culture analysis, on the surface of cholesterol gallstones^6^. Our study also suggested the presence of a biofilm on the surface of both pigmented and cholesterol type gallstones since stringent UV sterilisation initially resulted in a reduction of the yield of extracted genomic DNA by over 90%. Indeed the presence of a microbial biofilm on the surface of the patients’ gallstones was confirmed by taxonomic and functional metagenomics analysis. The following genera were identified in the gallstone metagenomes that harbor genes which are putatively associated to biofilm production.

#### Klebsiella

The *CsgD* gene is a transcriptional activator involved in the regulation of curli fimbriae biosynthesis^13^ (Table 4). Curli fimbriae have been identified to be significant EPS components within *Enterobacteriaceae* and involved in bacterial adherence to abiotic substances and cell adhesion during symbiotic or infectious processes^13^. *Klebsiella*, an enterobacterium, has exhibited the *CsgD* gene, alongside the Type 1 and Type IV fimbriae in this study. The Type 1 and Type IV fimbriae are part of the gene cluster *fim*, containing all the components required for fimbrial assembly, and associated with capsule and pilin processes - significant factors in colonisation and biofilm production^17^. Type 1 and Type IV fimbriae have also been shown to facilitate biofilm assembly on both abiotic and host-derived extracellular matrix protein surfaces^13^. The *wza* and *wzc* genes encode for surface molecules involved in capsule assembly and are considered to be important in the early stages of biofilm formation by *Klebsiella pneumoniae*^13,17^. A study isolating the genes involved in biofilm formation of the *K*. *pneumoniae* strain causing Pyogenic Liver Abscess found *SugE* an important gene that affects biofilm production by modulating capsular polysaccharide production and biofilm mucoviscosity^17^. The *ClpX* and *LuxR* regulatory genes, part of the sugar-specific phosphotransferase systems, and the cold shock protein *cspD* were also implicated in biofilm production^17^.

Recently, various strains of *Klebsiella* were tested to determine those with the highest biofilm production and the genes associated with this process^13^. The strain identified with the highest output of biofilm implicated the *RbsA* and *RbsC* genes alongside the quorum sensing molecule Autoinducer 2 (AI-2) and the prophage CP4–57 integrase as putitavively involved in the process^13^. These important genes associated with *Klebsiella* were also identified in our study (Table 4) The *RapA* gene identified has been shown to play a role in regulating the *yhcQ* gene that encodes a putative multidrug efflux pump and *yeeZ*, a gene associated with biofilm production^21^. Polyphosphate kinase has been linked with biofilm development, quorum sensing and virulence in *P*. *aeruginosa* and was also annotated to *Klebsiella* in our study^47,48^.

#### Escherichia

The surface molecule encoding *wzb* and *wzc* genes, important for capsule assembly and early stage biofilm formation in *K*. *pneumoniae*^13,16^, were also associated with *Escherichia* in our study (Table 4).

#### Enterococcus

Similar to genes involved in biofilm formation (the *RbsA*, *SugE*, *ClpX*, *LuxR*, *CspD*) discussed for *Klebsiella* above were associated with *Enteroccoccus* in our study. *Enterococcus* also exhibited the *galE* gene shown to influence lipopolysaccharide structure, colonisation and biofilm formation^49^.

#### Other relevant cellular processes

**Figure 4:**
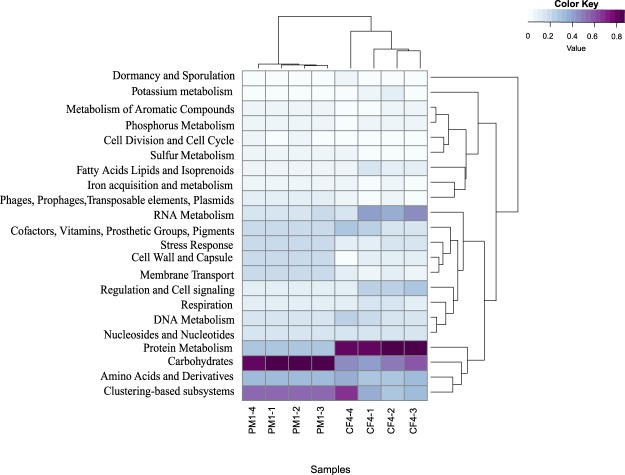
Heatmap with the major functional gene categories (acquired from the Subsystems, SEED Database) identified in the gallstones (n = 4) of patients PM1 (pigmented) and CF4 (cholesterol). The color key shows the relative abundance of the gene categories in the gallstones. The dendrograms illustrate the relationship between samples showing that the distribution of genes is relatively similar between replicate stones, but greatly differ between the two patients. For example, bacterial genes involved in the carbohydrate vs. protein metabolism were more abundant in gallstones from PM1 vs. CF4.

Consistent with a more prominent presence of number of species and genes encoding for processes associated with resistance to oxidative stress from bile and biofilm production, patient PM1 also exhibitied an overall higher abundance of genes involved in stress response, cell wall and capsule production, cluster-based subsystem activity as well as carbohydrate metabolism compared to patient CF4 (Fig. 4). A potentially enhanced carbohydrate metabolism in PM1 may be attributed to low levels of nutrients in the gallbladder resulting in microbes to metabolise excess biofilm (namely polysaccharides), as reported previously^50^. In contrast, Gram-positive bacteria, mainly associated with gallstones of patient CF4, reveal a higher relative abundance of genes involved in protein metabolism. One hyothesis is that these bacteria are involved in the decomposition of dead human cells associated with the gallstones of this patient (Fig. 4) as inferred from the high relative abundance of human genes compared to PM1. Another explanation for the observed diffence in a carbohydrate *vs*. protein dominated microbial metabolism between the gallstone types is that biofilms of Gram-negative and Gram-positive bacteria differ chemically from each other. EPS produced by Gram-negative bacteria, which predominate in PM1, exhibit anionic properties (attributed to uronic acids) that enable calcium and magnesium ions to bind with polymer strands providing a more tightly bound biofilm architecture^45^. Gram-positive bacteria (mainly in CF4) have been shown to exhibit a more cationic EPS charge, and be composed of teichoic acid mixed with small quantities of protein^45,51^, which may explain a higher relative abundance of genes involved in protein metabolism.

### Cholesterol analysis

We conducted compound specific isotopic analysis (CSIA) of the patients carbon (δ^13^C) and hydrogen (δD) isotopes of cholestrol to ascertain possible dietary or exogenous environmental factors that may be associated with or divergent from the bacteria identified in this study. Individual compounds in a complex mix (i.e. a gallstone made from patient-specific cholesterol/bile mixtures) can have distinct differences in their carbon, hydrogen, oxygen, nitrogen or sulfur isotopic signatures^52^. The marked difference between the isotopic weight of δ^13^C of an identified compound in two gallstones, for example, can indicate that a different source or mechanism was utilised during the synthesis of the compound^52^. We investigated whether or not the patients’ native stones were homogenous and if homogeneity existed between the patients themselves.

The only molecular component identified by GC-MS within the stones of each patient was the Cholest-5-en-3β-ol compound with trace amounts of 5α-Cholest-7-en-3β-ol. Subsequently, CSIA was conducted on the non-derivatised Cholest-5-en-3β-ol compound of each of the patients’ stones (Table 5).

**Table 5:** Compound Specific δ^13^C and δD results for the patients PM1 and CF4.

δ^13^ C (‰VPBD)				δ^D^ (‰VSMOW)			
PM1-1	−24.7(0.1)3	CF4-1	−23.1(0.1)3	PM1-1	−231(0)3	CF4-1	−252(0)3
PM1-2	−25.9(0.1)3	CF4-2	−23.5(0.1)3	PM1-2	−222(1)3	CF4-2	−253(0)3
PM1-3	−24.8(0.4)3	CF4-3	23.3(0)3	PM1-3	−218(3)3	CF4-3	254(0)3
PM1-4	−24.9(0.1)3	CF4-4	23.7(0)3	PM1-4	−221(1)3	CF4-4	254(1)3

The Cholest-5-en-3β-ol δ^13^C values obtained for patient PM1 showed a range between −24.7 and −25.9‰, and a range between −23.1 and −23.7‰ for patient CF4, resulting in an approximately 1‰ difference across the four stones analysed for each patient. The Cholest-5-en-3β-ol δD showed a value range of −218 and −231‰ for patient PM1, resulting in an approximately 13‰ difference across the four stones analysed for this patient. The Cholest-5-en-3β-ol δD showed a value range of −252 and −254‰ for patient CF4, resulting in a negligible 2‰ difference across the four stones analysed for this patient.

Within each patient both the δ^13^C and δD values for Cholest-5-en-3β-ol were not significantly different amongst the 4 stones analysed, supporting a common source for Cholest-5-en-3β-ol. Between the two patients the δ^13^C and δ D values differed only by minor amounts also supporting a common source for Cholest-5-en-3β-ol.

## Conclusions

This pilot study explored taxonomic and functional metagenomics and sterol homogeneity within two patients of diverse backgrounds to elucidate a possible universal factor at play in gallstone pathogenesis and formation. For the first time, functional genes were identified that were associated with bile stress response and biofilm development as possible microbial processes leading to the formation of both pigmented and cholesterol-type gallstones. In the analysed pigmented stones, genes involved in biofilm formation were mainly recovered from clinically pathenogenic *Klebsiella* and *Enterococcus* while bile resistance genes were present also in *Escherichia*, *Shigella*, *Serratia* and *Bacillus*. *Klebsiella* was also present in one of the chlolesterol gallstones, while the remaining analysed cholesterol stones showed a predominance of Gram-positive bacteria that were not identified within the pigmented stones. *Klebsiella* was also the only genus to exhibit DMSO respiration, giving it a distinct advantage in the anoxic environment of the human gallbladder. This, in conjuction with being the genus to exhibit the highest number of genes involved in bile stress response, and biofilm formation, may place *Klebsiella* as a major player in gallstone pathanogenesis. Further, pigmented stones, predominated by Gram-negative bacteria, revealed a high proportion of genes involved in carbohydrate metabolism, whilst cholesterol stones indicated a profile dominanted by protein metabolism. A possible explanation for the observed diffence in a carbohydrate vs. protein dominated microbial metabolism between the gallstone types is that biofilms of Gram-negative and Gram-positive bacteria differ chemically from eachother resulting with the latter having a higher protein content in the EPS matrix. Fungal and archaeal genes were not detected in both types of stones. Complementary carbon and hydrogen isotopic analyses of cholesterol within the patients’ stones revealed homogeneity, suggesting a common diet or cholesterol synthesis pathway that has only a minor influence on microbial composition.

This pilot study provides a framework to study microbial processes that play a potential role in gallstone formation across markedly different types of stones and patient backgrounds. In addition, future studies could also involve metatranscriptomic profiling to ultimately reveal which bacteria are actively expressing genes involved in processes such as bile stress response and biofilm formation that could contribute to the pathenogensis of gallstones.

## Methods

### Sample collection

Samples were collected whilst the patients were undergoing laproscopic cholecystectomy and were immediately rinsed in sterile saline solution (9 g L^−1^ NaCl) and placed in sterile glass containers. The samples were immediately stored at −80 °C until further processing. All patients provided written informed consent upon enrolment to the study. The study was designed with the aid of the National Health and Medical Research Council (NHMRC), according to the guidelines stipulated in the National Statement on Ethical Conduct in Human Research 2007^53^, and the Australian Code for the Responsible Conduct of Research 2007^54^. The study, which includes all associated experiments and methods, was approved by and met the ethical guidelines of the South Metropolitan Health Service Human Research Ethics Committee (HREC Reference: 15–136), the Fiona Stanley Human Research Ethics Committee (Ref: 2015–136), the St John of God Health Care Human Research Ethics Committee (Ref: 1021), and the Curtin University Human Research Ethics Committee (Ref: HR229/2015).

### DNA extraction

Genomic DNA of 4 gallstones from each patient were obtained from extractions following the procedure described by Haigh and Lee^9^. Inside a HEPA-filtered laminar flow bench, individual gallstones (~100 mg each) were pulverized using a heat-sterilized mortar (500 °C, 8 h). 700 uL of 1% SDS solution was added to each pulverized gallstone and incubated under rotation at room temperature for 12 h). Lithium chloride was added (final concentration of 1.5 M) following cell lysis through homogenisation in a FastPrep 96 Instrument (MP Biomedicals LLC, NSW, Australia) (1600 rpm, 60 sec). After centrifugation (5 min, 10,000 rcf) 1 vol of Phenol-Chloroform-isoamylalcohol 25:24:1 (PCI) pH 8 was added to the supernatant, vortexed for 1 min, and centrifuged for 5 min at 10,000 rcf. The PCI extraction was repeated once and 0.4 vol of molecular grade 80 vol% ethanol was added to the aqueous phase. The sample was then transferred to a Spin™ Filter and DNA was eluted from the filter using Solution C6 following the guidelines of the PowerSoil DNA Isolation Kit (Mo Bio Laboratories Inc, CA, U.S.A). PCR-inhibiting impurities were completely removed using the OneStep PCR Inhibitor Removal Kit (Zymo Research, U.S.A). The DNA concentration was quantified fluorometrically (NanoDrop 3300 Fluorospectrometer; Thermo Fisher Scientific, MA, U.S.A) using the Quant-iT PicoGReen dsDNA Assay kit (Life Technologies, VIC, Australia).

### Quantitative PCR

To quantify the amount of bacterial 16S rRNA gene copies, an aliquot of the extracted and purified genomic DNA was subjected to quantitative polymerase chain reaction (qPCR) using general primers^55^ targeting the V4 region of bacterial 16S rRNA. All reactions were performed using SYBR Premix Ex Taq (TLi RNase H Plus) (Takara Bio Inc) in a Realplex quantitative PCR cycler (Eppendorf) and involved initial denaturing (1 min at 95 °C), followed by 32 cycles including denaturing (5 s at 95 °C), primer annealing (30 sec at 60 °C), primer extension and imaging of newly formed fluorescent (SYBR^®^ green I labelled) double-stranded DNA (72 °C for 60 sec). Between 10^1^ and 10^6^ copies (10-fold dilution series) of bacterial 16S rRNA were added to reaction mixtures and served as standards during qPCR to calibrate the copy numbers of bacterial 16S rRNA in the gallstone samples.

### Metagenomic library preparation and sequencing

Metagenomic libraries were prepared using the NEBNext Ultra II DNA Library Prep Kit for Illumina (New England BioLabs Inc.) according to manufacturer’s instructions. The amplification involved 13–15 cycles. The resulting libraries were concentrated to a volume of 20 uL using Amicon Ultra centrifugal filer units Ultra-0.5 MWCO 30KDa. Gel electrophoresis (2 wt%, 50 min, 120 V) was performed with 10 uL of the concentrated libraries and gel fragments (200–500 bp) were excised and gel purified with the Monarch DNA Gel Extraction Kit (New England BioLabs Inc). The final volume after gel purification for each barcoded library (n = 8) was 20 uL and were sent to the Australian Genomic Research Facility (AGRF) in Perth, Western Australia for final quality checking and sequencing. At AGRF, the Illumina HiSeq. 2500 platform was used to generate 2 × 100-bp pair-end sequencing reads. The HiSeq Control Software (HCS) v2.2.68 and Real Time Analysis (RTA) v1.18.66.3 software performed real-time image analysis and base calling on the HiSeq instrument computer. The AGRF Illumina bcl2fastq 2.19.0.316 pipeline was used to generate the sequence data.

### Processing of sequence data and bioinformatics

Approximately 280 million paired-end sequence reads (see Supplementary Table S1) were imported into CLC Genomics Workbench 8.0 (CLC Bio) and trimmed of ambiguous reads to a quality limit of 0.5. Contigs were assembled using the CLC Genomics Workbench paired-end Illumina (*de novo*) read assembler with automatic bubble and word size, length fraction of 0.5, similarity fraction of 0.95, and a minimum contig size cut-off of 300 nucleotides. Contigs were assembled without scaffolding to reduce the formation of chimaeric assemblies. The CLC Genomics Workbench read mapping option was used to map reads onto contigs. ORFs within the contigs were detected using FragGeneScan^8^. Taxonomic assignments of contigs were performed using the NCBI BLASTp software suite against the SEED database of predicted proteins from cultivated microbial genomes with assigned taxonomy. The basis for taxonomic assignment of the ORFs was amino acid similarity of >60% over an alignment length of >50 amino acids to predicted proteins present in the database with an assigned taxonomy^8^.

A matrix showing the relative abundance (average coverage) of annotated ORFs deriving from specific taxa per sample was produced using a python script publicly available online (bitbucket.org/wrf), and was subsequently used for downstream analysis. Heatmaps were performed in R (http://www.r-project.org/) using the vegan (http://vegan.r-forge.r-project.org/) and the Bioconductor Heatplus (https://bioconductor.org/biocLite.R) package. The data was normalized, with the Hellinger function used to produce the taxonomy overview heatmap to show species that may have been obscured by the dominant reads (Fig. 3). The overview of Subsystems, Level 1, cellular processing category annotations were obtained from the SEED database *via* MG-RAST (Project ID: mgp81110–81111; metagenomics.anl.gov). The Primer-E software package (http://www.primer-e.com/) was used to generate principle coordinate analysis (PcoA) plots using the Bray-Curtis distance metric.

### Gas Chromatography Isotope Ratio Mass Spectrometry

Four gallstones from each patient were individually crushed in heat-sterilized (500 °C, 8 h) mortars. The grounded powder was then extracted *via* sonication (1 h) with dichloromethane (DCM) and methanol (9:1). The extracts were then fractionated by small-scale column liquid chromatography^56^. Approximately 2 mg of the total extract was placed on top of a small column (5 × 0.5 cm i.d.) of activated silica gel (160 °C, 8 h). The first hydrocarbon fraction was eluted with *n*-hexane (2 mL), the second hydrocarbon fraction with DCM in *n*-hexane (1:4, 2 mL), and the more polar fraction with an equal mixture of DCM and methanol (1:1, 2 mL). The fractions were analysed by gas chromatography-mass spectrometry (GC-MS).

The polar fractions (containing Cholest-5-en-3β-ol) were each analysed by compound specific isotope analyses to obtain δ^13^C and δD values of Cholest-5-en-3β-ol. The instrument used was a Thermo Delta V Advantage isotope ratio monitoring mass spectrometer (irMS), coupled to a Thermo Trace GC Ultra via a GC Isolink and Conflo IV. The column used was an Agilent DB-5MS Ultra-Inert, 60 m long, 0.25 mm (i.d.), with 0.25 µm film thickness. An aliquot of 1 µL of each fraction was injected into the split/splitless injector in splitless mode, held at 280 °C. The GC oven was increased from 40 to 325 °C at 10 °C/min, then held at 325 °C for 10 min. The carrier gas used was helium held at a constant flow of 1.5 mL/min.

For the carbon isotope analysis, GC column outflow was passed through the GC Isolink combustion reactor (copper oxide / nickel oxide, 1000 °C) to combust hydrocarbons to CO_2_. For hydrogen isotope analysis, the outflow passed through the high-temperature conversion reactor (graphite-lined, 1420 °C) and was pyrolysed to H_2_. The CO_2_ / H_2_ passed through the Conflo IV interface to the irMS, which measured m/z 44, 45 and 46 (for CO_2_) or m/z 2 and 3 (for H_2_). The δ^13^C and δD values were calculated from the measured masses by Thermo Isodat software, and calibrated to the VPDB (for CO_2_) and VSMOW (for H_2_) scales by comparison with a mixture of n-alkane standards of known isotopic composition.

### Data availability

Data are available as raw sequence reads from the NCBI Short Read Archive (SRA) under accession number SRP136827 and as assembled contigs in MG RAST (metagenomics.anl.gov) under accession numbers 4754155.3, 4754325.3, 4754326.3, 4754607.3, 4754608.3, 4754609.3, 4754610.3, 4754611. Fasta files containing the expressed ORFs with signal peptides are available from the authors upon request.

## Electronic supplementary material

 Supplementary Information
