# Author Correction: Metagenomics of pigmented and cholesterol gallstones: the putative role of bacteria

**DOI:** 10.1038/s41598-020-60081-8

**Published:** 2020-03-04

**Authors:** S. H. Kose, K. Grice, W. D. Orsi, M. Ballal, M. J. L. Coolen

**Affiliations:** 1 School of Molecular and Life Sciences, Curtin University, Perth, WA 6102 Australia; 2 WA-Organic and Isotope Geochemistry Centre, School of Earth and Planetary Science, Curtin University, Perth, WA 6102 Australia; 3 Department of Earth and Environmental Science, Paleontology and Geobiology, Ludwig-Maximilians-Universität München, 80333 Munich, Germany; 4 GeoBio Centre LMU, Ludwig-Maximilians-Universität München, 80333 Munich, Germany; 5 Fiona Stanley Hospital, 11 Robin Warren Dr, Murdoch, 6150 WA Australia; 6 St John of God Murdoch Hospital, Barry Marshall Parade, Murdoch, 6150 WA Australia

Correction to: *Scientific Reports* 10.1038/s41598-018-29571-8, published online 25 July 2018

The original version of this Article contained errors. Affiliation 1 and Affiliation 2 were incorrectly given as ‘School of Molecular and Life Sciences, W.A Organic and Isotopic Geochemistry Centre, Curtin University, Perth, WA, 6000, Australia’ and ‘Department of Earth and Environmental Science, GeoBio Centre LMU, Ludwig-Maximilians-Universität München, 80333, Munich, Germany’ respectively. In addition, two affiliations were omitted, which are now listed as Affiliations 2 and 4.

Furthermore, K. Grice and M. J. L. Coolen were incorrectly affiliated with ‘School of Molecular and Life Sciences, W.A Organic and Isotopic Geochemistry Centre, Curtin University, Perth, WA, 6000, Australia’.

The correct affiliations list and affiliated authors are given below.

Affiliation 1:

School of Molecular and Life Sciences, Curtin University, Perth, WA 6102, Australia.

S.H. Kose

Affiliation 2:

WA-Organic and Isotope Geochemistry Centre, School of Earth and Planetary Science, Curtin University, Perth, WA 6102, Australia.

S.H. Kose, K. Grice, M.J.L. Coolen

Affiliation 3:

Department of Earth and Environmental Science, Paleontology and Geobiology, Ludwig-Maximilians-Universität München, 80333, Munich, Germany.

W.D. Orsi.

Affiliation 4:

GeoBio Centre LMU, Ludwig-Maximilians-Universität München, 80333, Munich, Germany.

W.D. Orsi.

Affiliation 5:

Fiona Stanley Hospital, 11 Robin Warren Dr, Murdoch, 6150, WA, Australia.

M. Ballal.

Affiliation 6:

St John of God Murdoch Hospital, Barry Marshall Parade, Murdoch, 6150, WA, Australia.

M. Ballal.

These errors have now been corrected in the PDF and HTML versions of the paper, and in the accompanying Supplementary Information file.

