# Supplementary material for: Metagenomics of pigmented and cholesterol gallstones: the putative role of bacteria
Source: Sci Rep. 2018 Jul 25;8:11218. doi: 10.1038/s41598-018-29571-8 (PMC6060111; doi:10.1038/s41598-018-29571-8)
Supplement: Supplementary file 1 — Supplementary Information [file 41598_2018_29571_MOESM1_ESM.pdf]

Metagenomics of pigmented and cholesterol gallstones: the putative role of bacteria

S.H. Kose[1,2]*, K. Grice[2], W.D. Orsi[3,4], M. Ballal[5,6] and M.J.L. Coolen[2]

[1] School of Molecular and Life Sciences, Curtin University, Perth, WA 6102, Australia

[2] WA-Organic and Isotope Geochemistry Centre, School of Earth and Planetary Science, Curtin University, Perth, WA 6102, Australia

[3] Department of Earth and Environmental Science, Paleontology and Geobiology, Ludwig-Maximilians-Universität München, 80333, Munich, Germany

[4] GeoBio Centre LMU, Ludwig-Maximilians-Universität München, 80333, Munich, Germany

[5] Fiona Stanley Hospital, 11 Robin Warren Dr, Murdoch, WA, 6150, Australia

[6] St John of God Murdoch Hospital, Barry Marshall Parade, Murdoch, WA 6150, Australia

*Corresponding author: sureyya.kose@curtin.edu.au

Table S1: Sequence Data Summary

	Pigmented Stones (N=4)	Cholesterol Stones (N=4)	Total (N=8)
Total Paired-End Sequence Reads	262,254,326	19,347,392	281,601,718
Paired-End Sequence Reads per sample	65,563,582 ± 26.5 10^6	4,836,848 ± 1.16 10^6	70,400,430 ± 30.0 10^6
Total ORFs	22,393,091	392,392	22,785,483
ORFs per sample	5,598,273 ± 2.75 10^6	98,098 ± 41.1 10^3	5,696,371 ± 2.75 10^6
